# Neural substrates of successful working memory and long-term memory formation in a relational spatial memory task

**DOI:** 10.1007/s10339-016-0772-7

**Published:** 2016-06-27

**Authors:** Heiko C. Bergmann, Sander M. Daselaar, Guillén Fernández, Roy P. C. Kessels

**Affiliations:** 1 Donders Institute for Brain, Cognition and Behaviour, Radboud University, Nijmegen, The Netherlands; 2 Department of Cognitive Neuroscience, Radboud University Medical Center, Nijmegen, The Netherlands; 3 Department of Medical Psychology, Radboud University Medical Center, Nijmegen, The Netherlands; 4 Neuropsychology and Rehabilitation Psychology, Radboud University, Montessorilaan 3, 6525 HR Nijmegen, The Netherlands

**Keywords:** Spatial memory, Working memory, Episodic memory, Neuroimaging, Subsequent memory

## Abstract

Working memory (WM) tasks may involve brain activation actually implicated in long-term memory (LTM). In order to disentangle these two memory systems, we employed a combined WM/LTM task, using a spatial relational (object-location) memory paradigm and analyzed which brain areas were associated with successful performance for either task using fMRI. Critically, we corrected for the performance on the respective memory task when analyzing subsequent memory effects. The WM task consisted of a delayed-match-to-sample task assessed in an MRI scanner. Each trial consisted of an indoor or outdoor scene in which the exact configuration of four objects had to be remembered. After a short delay (7–13 s), the scene was presented from a different angle and spatial recognition for two objects was tested. After scanning, participants received an unexpected subsequent recognition memory (LTM) task, where the two previously unprobed objects were tested. Brain activity during encoding, delay phase and probe phase was analyzed based on WM and LTM performance. Results showed that successful WM performance, when corrected for LTM performance, was associated with greater activation in the inferior frontal gyrus and left fusiform gyrus during the early stage of the maintenance phase. A correct decision during the WM probe was accompanied by greater activation in a wide network, including bilateral hippocampus, right superior parietal gyrus and bilateral insula. No voxels exhibited supra-threshold activity during the encoding phase, and we did not find any differential activity for correct versus incorrect trials in the WM task when comparing LTM correct versus LTM incorrect trials.

## Introduction

 The underlying neural substrate of working memory (WM) is still under debate. “Classical” dual-process theories implied frontal as well as parietal regions as being critical for the processing and maintenance of a limited amount of information (supposed to be within WM capacity) across a short interval. More recent accounts suggested to distinguish memory systems based on the underlying processing operations required to successfully complete the task at hand, rather than on the interval between study and test (Jonides et al. 2008; Konkel and Cohen 2009; Ranganath and Blumenfeld 2005). In this view, the exact task characteristics as well as how the task is typically executed should be concisely defined and analyzed a priori. For instance, it has been argued that tasks which require the rapid encoding of associations would engage the hippocampus—a brain region argued to be not involved in working memory function (Jeneson and Squire 2012)—irrespective of the length between study and test and whether the stimuli had been processed consciously or not in the first place (Henke 2010). This is most likely due to the anatomical characteristics and extensive reciprocal connectivity of the hippocampus with polymodal neocortical association areas (Suzuki and Amaral 1994), serving as a hub of brain network communication for memory (Battaglia et al. 2011).

The latter proposal is in line with the increasing amount of evidence suggesting hippocampal involvement not only in (episodic) long-term memory (LTM), but also in relational WM tasks, in patient studies (Crane and Milner 2005; Giovanello et al. 2003; Hannula et al. 2006; Hartley et al. 2007; Holdstock et al. 1995; Nichols et al. 2006; Olson et al. 2006a, b; Piekema et al. 2007; Rose et al. 2012; Turriziani et al. 2004; however, see Jeneson et al. 2010, 2011, 2012; Stark et al. 2002; Stark and Squire 2003), intracranial EEG and MEG studies (Axmacher et al. 2008, 2010a, b) as well as functional neuroimaging studies (Axmacher et al. 2008, 2009; Hannula and Ranganath 2008; Kirwan and Stark 2004; Luck et al. 2010; Nichols et al. 2006; Olsen et al. 2009; Oztekin et al. 2009; Piekema et al. 2006, 2009, 2010; Ranganath et al. 2005; Schon et al. 2009). However, while the aforementioned studies highlighted an important role for the hippocampus in the execution of WM *tasks,* this does not necessarily imply that the performance on these paradigms solely relies on WM *processing*. In previous studies (Bergmann et al. 2012, 2015), we argued that performance of WM tasks is also supported by (incidental) LTM processes, even when memory is tested only seconds after learning (cf. Jeneson and Squire 2012). That is, people may use mnemonic strategies during WM paradigms, such as semantic coding, which rely on LTM rather than WM. Consequently, WM tasks may recruit brain regions that are more typically associated with LTM (cf. Baddeley 2012).

In order to identify the brain areas supporting the successful execution of associative WM tasks, we developed a paradigm consisting of a delayed-match-to-sample (WM) task, assessed in a event-related functional MRI study and an unexpected delayed recognition memory (LTM) task outside the scanner, testing the same (pairs of) stimuli as during the WM task (that is, task characteristics were held constant across the two memory tests; Bergmann et al. 2012, 2015). Subsequent memory effects were analyzed for both the WM and the LTM tasks, by contrasting hits with misses on either memory task. Critically, when assessing the “subsequent WM effect,” analyses concentrated exclusively on stimulus pairs that were not correctly recognized in the subsequent LTM task. As there is no (successful) LTM representation for these trials, this reduces the confounding factor of incidental LTM formation during the execution of a WM task. This paradigm provided initial insight into the underlying neural substrates of successful associative WM, using a non-spatial WM and LTM tasks. Importantly, we showed that hippocampal involvement during the encoding phase of the WM task was associated with successful LTM formation. Hippocampal activation was not found for stimuli that were not remembered correctly in the long term, but that were successfully maintained in the WM task (Bergmann et al. 2012).

One alternative explanation for this absent finding of hippocampal involvement during “pure” WM processing may lie in the task characteristics. That is, while our paradigm was associative (i.e., combinations of faces and houses had to be maintained in WM and subsequently retrieved in the LTM part of the paradigm), it was not spatial in nature. Possibly, the use of a relational spatial (working) memory paradigm may result in hippocampal activation already during the WM stage (cf., Piekema et al. 2006) even when the information is not remembered in the long term. Hannula and Ranganath (2008) also argued that many studies failing to demonstrate hippocampal involvement typically used paradigms that may not always required relational memory processing. To overcome this, Hannula and Ranganath (2008) therefore employed a challenging object-location short-term memory task with a clear allocentric spatial component. In their analysis (contrasting correct with incorrect trials), they found, among others, increased hippocampal activation for correct versus incorrect trials for both the encoding and the probe phase. However, it could not be determined to what extent this was related to incidental LTM formation rather than “true” WM processing, since the authors administered only a short-term memory task.

To investigate whether an (allocentric) spatial WM tasks would result in hippocampal involvement even in the absence of successful LTM formation, we adopted our combined WM and LTM paradigm. In a functional MRI study, we determined the underlying neural substrates of successful spatial WM and LTM. In each trial, we presented a rendered scene (indoor or outdoor scenes) in which trial-unique objects were placed. Subsequently, during the probe phase, the scene was shown from a different angle and the objects were either presented at the same spot or one object changed its location or two objects swapped their location. On completion of the WM task, we administered an unexpected recognition memory task outside the scanner to assess LTM for the object-location mappings. We hypothesized that (1) already during the WM phase of the task, successfully maintained object locations would require hippocampal processing compared to object locations that were not correctly maintained during the WM phase and (2) this hippocampal involvement was independent from the LTM success. That is, hippocampal involvement during WM maintenance was expected to be present both for later remembered and later forgotten object locations and would not predict LTM success.

## Methods

### Participants

Thirty right-handed healthy undergraduate students (12 men; mean age = 20.6 years, ranging from 18 to 27 years) participated in the study. However, five participants (one man) were excluded from further analyses because they performed at chance level on the LTM task and another participant (a woman) performed on chance level (proportion correct 55.7) on the WM task. The remaining 24 participants (mean age = 20.7 years) all had normal or corrected-to-normal vision. None had a history of neurological, major medical, or psychiatric disorders. Participants gave written informed consent according to the local ethics committee (CMO Region Arnhem-Nijmegen) and the declaration of Helsinki.

### Behavioral task

**Fig. 1:**
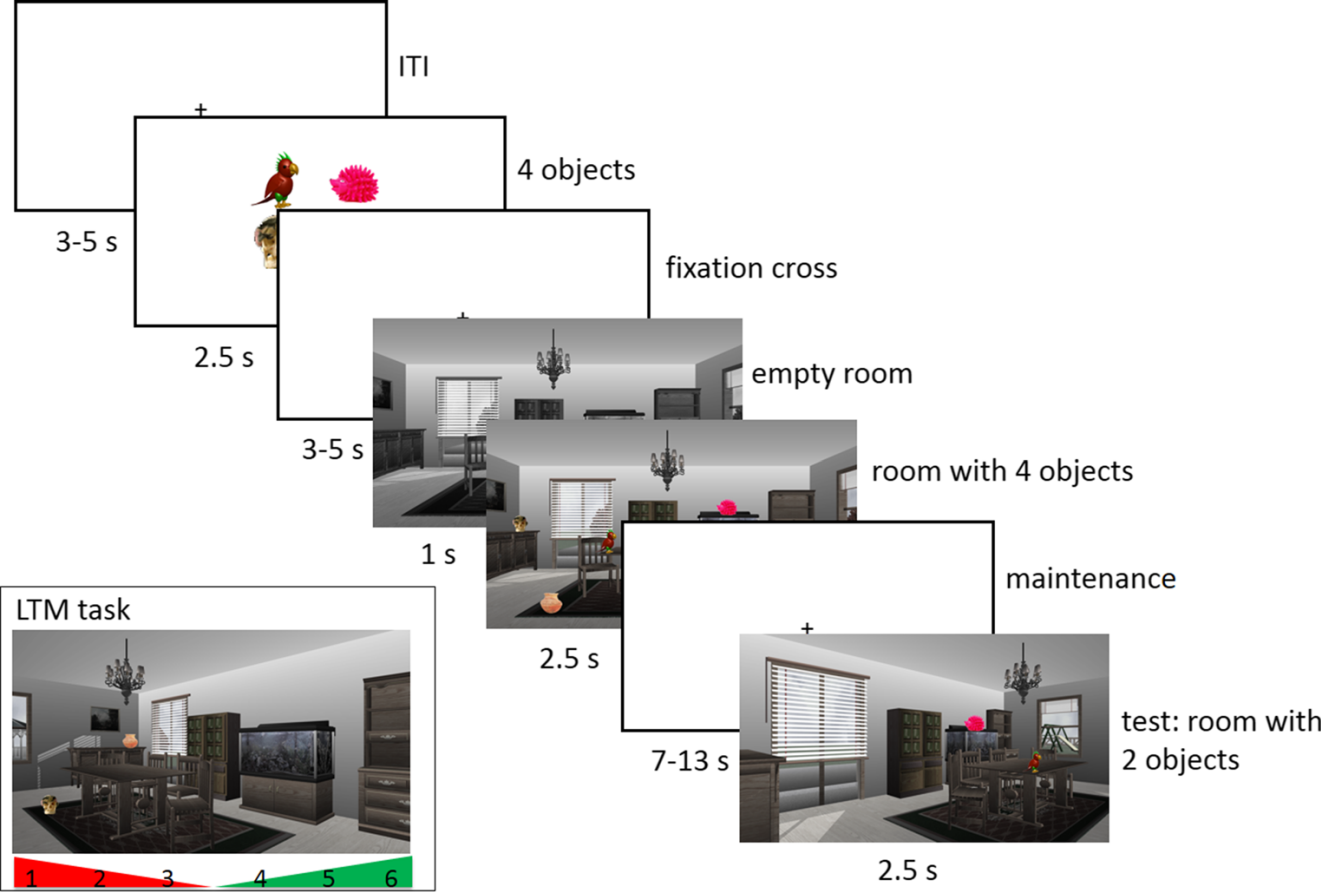
Schematic overview of one trial of the delayed-match-to-sample (WM) task and the LTM task. In each trial, the four objects that were to be placed in the scene were first presented. Subsequently, the room without the objects was shown, whereupon the four objects were placed. During the probe phase, the room was shown from a different angle and subjects had to indicate whether the two objects were at the same spot as during the learning phase or not. In the LTM task, the two other items were probed and the room was again presented from a different orientation

An object-location delayed-match-to-sample memory task (hereafter referred to as WM task) was administered in an MRI scanner. The task consisted of an (extended) encoding phase, a maintenance phase and a probe phase (see Fig. 1 for a schematic overview of one trial of the task). In total, 140 trials were presented in the scanner. In each trial, the positions of objects that were placed in indoor and outdoor scenes had to be remembered. The to-be-remembered objects were typical everyday objects which could easily be named, like a candle, a ball, a cup, etc.

The encoding phase started with the presentation of four objects which were shown on a white background for 2.5 s. This was followed by a variable interstimulus interval of 3–5 s (in steps of 0.5 s). Subsequently, one of fourteen rendered scenes, created with Punch! Home Design software (sized 720 × 406 pixels), was presented. These fourteen scenes all had similar dimensions and depicted clearly distinct scenes, for instance a kitchen, a bathroom and a garage (a label was depicted underneath the scene to help participants discriminating between scenes). Each scene had unique furniture and appliances. The to-be-remembered objects were shown in the scenes and, to this end, 12 possible object locations (i.e., coordinates) were defined for each scene. Each scene was shown for 1 s. Subsequently, the four previously shown objects were pseudo-randomly placed in four of the twelve pre-defined locations of the respective scene and shown for 2.5 s (the objects were placed 1 s after presentation of the scene to reduce the participants’ visual scanning and make use of a visual pop-out effect). The encoding phase was followed by a variable 7- to 13-s maintenance interval, randomly varied in steps of 2 s.

During the probe phase, the same scene was shown again for 1 s. However, the scene was now depicted from a different angle. This location shift was randomly determined for each trial (pseudo-randomized, 50 % left, 50 % right shifts). Subsequently, two of the previously shown four objects were placed in the scene again. In 50 % of the trials, these two objects were placed in the same location as during the encoding phase (match trial), in 25 % of the trials only one of the two objects changed its location, and in the remaining 25 % the two objects swapped their positions. The participant’s task, however, was only to indicate whether the two objects were placed at the same positions as during the encoding phase (“match”) or not (“non-match”). A response had to be given within the allotted time constraint of 2.5 s by pressing the left button with the right index finger (“match”) or the right button with the right middle finger (“no match”) using an MR-compatible keypad. Preceding the experiment, participants received written instructions and completed eight practice trials outside the scanner to get familiarized with the task.

After scanning, participants were presented with an unexpected recognition memory test (hereafter referred to as LTM task) to assess LTM for the object locations that were shown in the scanner. This task was highly similar to the probe phase of the WM task. That is, each of the 140 trials started with the presentation of the scene for 1 s and was followed by the placement of two objects. However, the location of the “camera” was again changed (i.e., if the location of the camera had been at the left side during the WM probe, it now was at the right side) and the two previously unprobed objects were tested (this was done to avoid double encodings). In 50 % of the trials, the two objects were placed in the same location as during the encoding phase (“match”), in 25 % on of the two objects changed its location and in the remaining 25 % the two objects swapped their positions. Again, participants only had to indicate if the location of the objects matched their original position (“match”) or not (“non-match”). In addition, participants could give a confidence rating that ranged from 1 (“definitely not at the same location”) to 6 (“definitely at the same location”). Figure 1 gives a schematic overview of the WM and LTM tasks.

### Image acquisition and data preprocessing

Images were collected with a 1.5-T Avanto MRI scanner system (Siemens Medical Systems, Erlangen, Germany) using a 32-channel radiofrequency head coil. First, high-resolution anatomical images were acquired using a T1-weighted 3D MPRAGE sequence (TR = 2250 ms, TE = 2.95 ms, flip angle = 15°, 176 sagittal slices, acquisition matrix = 256 × 256, FOV = 256 mm, voxel size = 1 × 1 × 1 mm^3^). Whole-brain functional images were collected using a T2*-weighted EPI sequence (TR = 2280 ms, TE = 40 ms, image matrix = 64 × 64, FOV = 212 mm, flip angle = 90º, slice thickness = 3.0 mm, distance factor = 10 %, voxel size 3.3 × 3.3 × 3.0 mm^3^, 32 axial slices). The first five volumes of the EPI series were excluded from the analysis to allow the magnetization to approach a dynamic equilibrium. Data processing started with realignment of the functional EPI-BOLD images, using a six-parameter, rigid-body transformation algorithm. Subsequently, the mean of the functional images was co-registered to the structural MR image using mutual information optimization. Functional images were then spatially normalized, resampled to create 3-mm isotropic voxels and transformed into a common stereotactic space, as defined by the SPM5 MNI T1 template. Finally, the images were spatially smoothed with an 8-mm FWHM Gaussian filter. Low-frequency drifts in the time domain were removed by modeling the time series for each voxel by a set of discrete cosine functions to which a cutoff of 128 s was applied.

### Data analysis

#### fMRI data analysis

The fMRI data were analyzed with statistical parametric mapping using SPM5 software (Wellcome Department of Cognitive Neurology, London). Subject-level statistical analyses were performed using the general linear model (GLM). We investigated which brain regions could predict success on the WM and LTM tasks during the encoding, maintenance phase as well as the WM probe phase. Based on memory performance, trials were divided into different categories. Participants could respond correctly (hits and correct rejections) and incorrectly (misses and false alarms) on both the WM and LTM tasks; four response categories were possible: (1) WM correct/LTM correct (in the remainder: WM+/LTM+), (2) WM correct/LTM incorrect (WM+/LTM−), (3) WM incorrect/LTM correct (WM−/LTM+) and (4) WM incorrect/LTM incorrect (WM−/LTM−). However, the combination WM−/LTM+ did not occur frequently, resulting in inadequate statistical power to be reliably estimated and therefore this combination was entered as a regressor of no-interest. The remaining three categories were entered as separate regressors of interest, as a function of the WM phase. In addition, the object presentation was also entered as a regressor of interest.

**Fig. 2:**
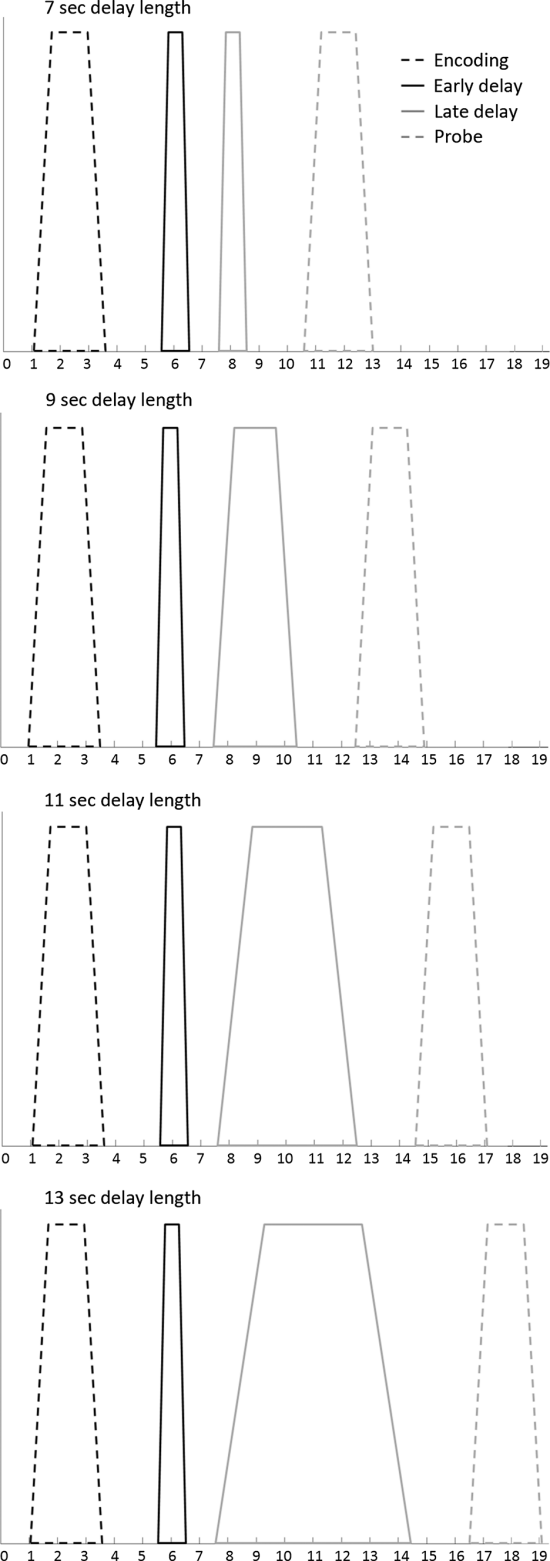
Vectors of expected neural activity corresponding to encoding, early and late delay and probe phase. Covariates modeling BOLD response on each WM trial were constructed by convolving the different stages (i.e., early delay, late delay or probe phase) with its respective duration and convolved with the canonical hemodynamic response function

The identical vector definition (i.e., onset, duration and expected neural activity associated with each component) as implemented by Ranganath et al. (2005) was used (see Fig. 2): the construction of the covariates for early and late stage of WM maintenance was based on the assumption that processing associated with the early stage would occur during the first few seconds of the maintenance phase. Processing associated with the late stage of WM maintenance, in contrast, was suggested to persist throughout the remainder of the WM maintenance phase. To minimize the possibility that activity associated with one particular WM stage was confounded with one of the other WM stages, onset and offset of the early and late stage of the delay phase were spaced apart from each other as well as from the probe phase (see Fig. 2).

#### Second-level analyses

The described individual contrast images were created and submitted to a second-level factorial analysis, consisting of two factors: (1) Phase, consisting of four levels (encoding, early delay, late delay and probe phase) and (2) Response Category, comprising the three levels of interest (WM−/LTM−, WM+/LTM− and WM+/LTM+). Participants were treated as random variable. Results from the random effects analyses were first thresholded at *p* = .001 (uncorrected). Subsequently, cluster size statistics were used as the test statistic. For whole-brain analyses, clusters at *p*_FWE_ < 0.05 (FWE corrected for multiple non-independent comparisons; Worsley et al. 1996) were considered significant and are reported together with the MNI coordinates of their local maximum. In addition, given the disputed role of the medial temporal lobe, an anatomical region of interest (ROI) was created which bilaterally covered the hippocampus or the parahippocampal region, respectively. These were used as a mask for small-volume corrections (tested at *p*_SVC_ < 0.05).

## Results

### Behavioral data

#### Working memory task

Mean hit rate was 76.01 % (±9.01) and mean false alarm rate 15.00 % (±7.86), *d*′ = 1.83, ± 0.47. Participants failed to respond within the time constraint of 2 s in 5.65 % of the trials.

#### Long-term memory task

**Fig. 3:**
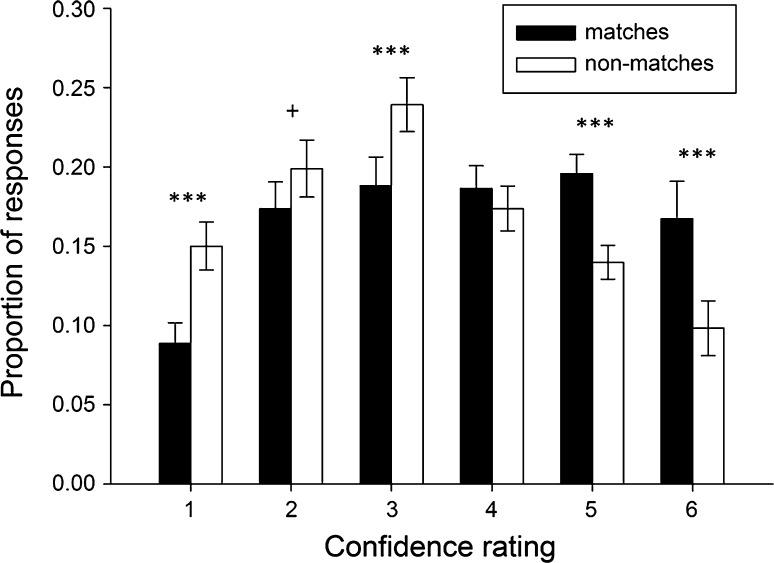
Behavioral performance on the LTM task. Distributions of mean hit and false alarm rates: Mean (±SEM) proportions of responses are depicted on the *y*-axis and confidence ratings (“*1*”: definitely a non-match; “*6*”: definitely a match) on the *x*-axis. ***p ≤ .001, +*p* = .056

Figure 3 shows the distribution of averaged response proportions in the LTM task. A 2 (stimulus type: match vs. re-arranged pair) by 6 (confidence rating: 6-point scale) repeated-measure MANOVA revealed an interaction between confidence rating and stimulus type, *F*(5, 102) = 14.80, *p* < .0005, *η*_p_^2^ = .39. Post-hoc paired sample *t* tests showed that the proportion of “6” [*t*(23) = 4.98, *p* < .0005] and “5” [*t*(23) = 4.00, *p* < .0005) ratings was significantly higher for matches than for non-matches. In contrast, the proportion of “1” [*t*(25) = 4.01, *p* = .001], “2” [*t*(23) = 2.02, *p* = .056], and “3” [*t*(23) = 4.33, *p* < .0005] ratings for matches was significantly lower than for non-matches (note that for the “2” ratings only a nonsignificant trend was observed). Finally, the proportion of “4” ratings did not differ between these two [*t*(23) = 1.24, *p* = .23]. These results demonstrate that participants were able to successfully discriminate between matches and non-matches at all confidence levels, except level 4. Consequently, “correct” LTM trials were defined as correctly endorsing an intact arrangement with a confidence rating of 5 or 6 and as correctly rejecting a rearranged arrangement with a confidence rating of 1, 2 or 3. In contrast, LTM were classified “incorrect” when participants failed to endorse intact pairs with a confidence rating of 5 or 6 or failed to reject a rearranged arrangement with a rating of 1, 2 or 3. Each participant had more than 10 events of each response category.

### Functional imaging data

#### Subsequent WM memory effect equating for LTM performance

##### Encoding phase

To control for possible contamination effects of LTM when assessing WM effects, we subsequently examined which brain regions were specifically recruited for correct WM trials as opposed to incorrect WM trials, when there was no evidence of successful LTM formation, i.e., WM+/LTM− > WM−/LTM−. No voxels showed significant BOLD signal changes for this contrast.

##### Early and late maintenance phase

**Fig. 4:**
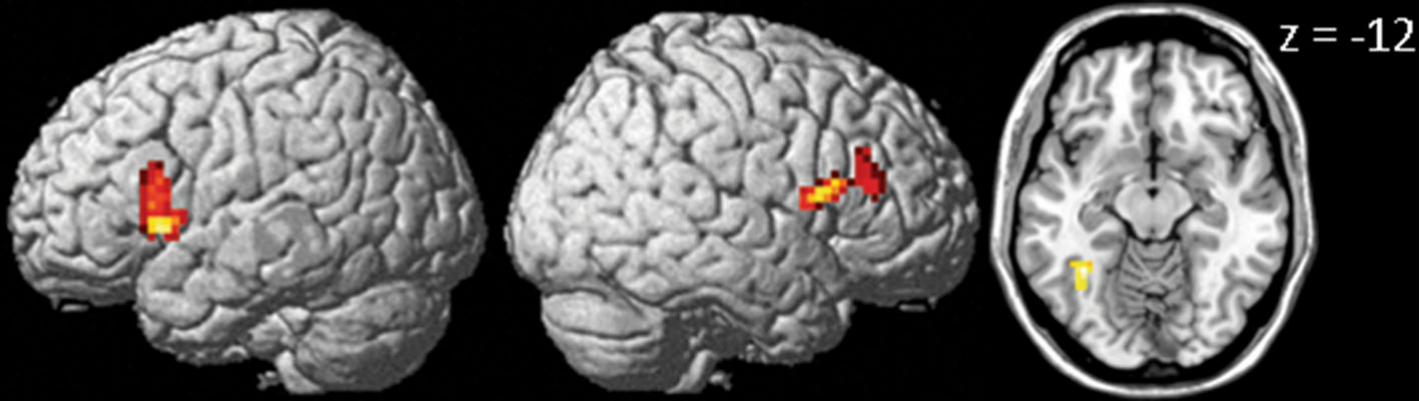
Brain areas related to successful WM processing during the early WM maintenance phase, equated for LTM performance (WM+/LTM− > WM−/LTM−). A correct WM decision was associated with greater activation in *left* and *right* inferior frontal gyrus and *left* fusiform gyrus. Activation clusters (*p* < .001, uncorrected, >30 voxels) superimposed on averaged (*n* = 24) high-resolution T1-weighted images

For the early maintenance phase (see Table 1 and Fig. 4), this analysis revealed greater activation in the left (local maximum at [−60, 15, 18]; *p*_FWE_ < .001) and right (local maximum at [51, 9, 12]; *p*_FWE_ < .001) inferior frontal gyrus. In addition, marginally significantly greater activation was found in the left fusiform gyrus (local maximum at [−36, −51, −12]; *p*_FWE_ = .069). Small-volume corrections for the medial temporal lobe did not reveal additional activation clusters. A similar analysis was performed for the late delay phase. However, no voxels exhibited supra-threshold activation.

**Table 1:** Activations for the subsequent WM effect equating for LTM performance (WM+/LTM− > WM−/LTM−) during (1) encoding, (2) early or (3) late stage of the WM maintenance phase and (4) probe

Brain region	BA	Cluster size	*t* value	*z* value	MNI		
					*x*	*y*	*z*
(1) Encoding—no supra-threshold clusters							
(2) Early delay							
Left inferior frontal gyrus	L 44	120	4.39^a^	4.30	−60	15	18
			3.85	3.79	−39	9	0
			3.79	3.73	−48	15	0
Right inferior frontal gyrus	L 44	91	4.45^a^	4.35	51	9	12
			4.21	4.12	57	33	18
			4.01	3.93	60	15	15
Left fusiform gyrus	L 37	42	4.82^a^	4.70	−36	−51	−12
(3) Late delay—no supra-threshold clusters							
(4) Probe							
Left hippocampus		5	3.77^b^	3.71	−36	−12	−18
Right hippocampus		4	4.30^b^	4.21	30	−6	−18
Left precentral/postcentral gyrus	L 4/5	244	4.54^a^	4.43	−21	−27	54
			4.28	4.19	−21	−27	69
			4.10	4.02	6	−6	48
Right postcentral gyrus/right superior parietal gyrus	L 2/5	300	4.86^a^	4.74	24	−24	66
			4.58	4.47	18	−48	66
			4.43	4.34	27	−39	57
Left middle occipital gyrus	L 37	88	4.53^a^	4.43	−45	−63	6
Left insula/operculum		103	4.24^a^	4.16	−39	−6	−18
			4.18	4.10	−33	−12	6
			3.76	3.70	−30	−3	0
Right insula/putamen		250	5.50^a^	5.32	27	−6	−3
			4.62	4.51	30	−3	12
			4.52	4.41	30	−3	−18

^a^ *p*_FWE_ < .05

^b^ *p*_SVC_ < .05

##### Probe phase

**Fig. 5:**
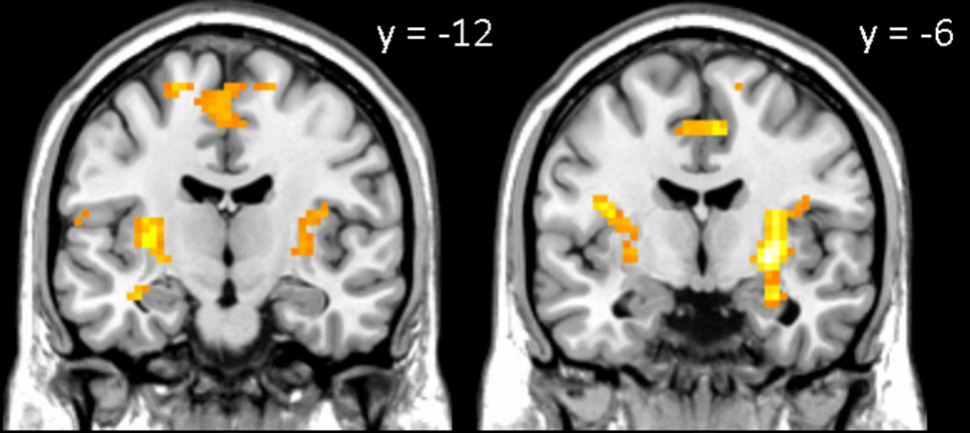
Brain areas associated with a correct WM decision during the WM probe phase, equated for LTM performance (WM+/LTM− > WM−/LTM−). Greater activation in *left* and *right* hippocampus, insula and bilateral post-central gyrus, extending into parietal lobe was found. Activation clusters (*p* < .001, uncorrected, >30 voxels) superimposed on averaged (*n* = 24) high-resolution T1-weighted images

Outside the medial temporal lobe, we found greater probe-related activation in the right post-central gyrus/superior parietal gyrus (local maximum at [24, −24, 66]; *p*_FWE_ < .001) as well as in the left precentral/postcentral gyrus (local maximum at [−21, −27, 54]; *p*_FWE_ < .001), the left middle occipital gyrus (local maximum at [−45, −63, 6]; *p*_FWE_ = .003), and left insula (local maximum at [−39, −6, 18]; *p*_FWE_ = .001) and a big cluster comprising the right insula and putamen (local maximum at [27, −6, −3]; *p*_FWE_ < .001). See Table 1 and Fig. 5 for details. Within the medial temporal lobe, this analysis revealed greater left (local maximum at [−36, −12, −18]; *p*_SVC_ = .005) and right (local maximum at [30, −6, −18]; *p*_SVC_ = .001) hippocampal activation for correct versus incorrect trials.

#### Subsequent LTM effect equating for WM performance

For the LTM task, we investigated which brain regions predicted successful LTM when pairs had already been correctly classified in the WM task. To this end, trials correctly recognized in WM and remembered in the LTM task were contrasted with stimulus sets recognized correctly in WM but not correctly in the LTM task (i.e., WM+/LTM+ > WM+/LTM−). Somewhat surprisingly, though, we did not obtain any supra-threshold activations for any of the four analyzed stages (encoding, early and late delay, probe phase).

## Discussion

The present paper investigated the underlying neural substrates of successful spatial relational WM and LTM. Critically, subsequent memory effects for both WM and LTM were “corrected” to minimize the potential confounds of either memory system. Since most previous studies investigated WM or LTM in isolation, they were unable to determine to what extent their reported findings might have been related to other memory processes or systems than those being formally under investigation. Hence, our study was based on the underlying rationale that LTM or processes more typically related to LTM may support the performance on a WM task, irrespective of the delay between study and test (and also irrespective of memory load). For four different stages of the WM task (encoding, early delay, late delay and probe phase), we assessed which brain regions were associated with either a successful decision on the WM task or the LTM task. WM and LTM will be discussed in turn in the following sections.

### Working memory task

The subsequent WM analysis for the encoding phase did not yield differential activity. This is particularly interesting as our previous study that focused on the encoding phase in a non-spatial associative WM task clearly demonstrated differential activity for both the subsequent WM and LTM effects (Bergmann et al. 2012). In that study, we found activation in content-specific visuo-perceptual areas being associated with a correct decision on the WM task. More specifically, we reported greater activation in the parahippocampal gyrus and fusiform gyrus, reflecting the fact that we used pairs of houses and faces as stimuli. This was explained by increased (or more efficient) content processing of (some of) the visual features of the presented stimuli (Bergmann et al. 2012). However, the stimuli in the present study did not belong to one particular category; many different stimuli were used that are thought to be processed in different areas of the brain. Hence, if one assumes that successful visuo-perceptual processing is critical for successful WM processing, particularly during the encoding phase, this could explain why we did not find stimulus-specific differential activity for correct versus incorrect trials in our present study. However, the primary task of the participants was to learn and remember the spatial configuration of each stimulus set, and by presenting the scene from a different angle we aimed to tap allocentric spatial processing. We hypothesized to extend previous findings that encoding-related activity in the hippocampus would predict success on the WM task (Hannula and Ranganath 2008), but we could not replicate this finding using the current paradigm. Possibly, some idiosyncratic feature of our paradigm might have obviated true relational memory processing (e.g., some participants indicated that they tried to encode the stimuli by their color and the order in which they were presented in the scene, which, however, does not appear to be a helpful strategy when the scene was rotated).

No supra-threshold activation was detected for the late delay stage, but greater activation in bilateral inferior frontal gyrus for correct versus incorrect trials was found for the early delay stage. Interestingly, in a previous study (Bergmann et al. 2012), we found encoding-related activity in a highly overlapping brain area (left inferior frontal gyrus), predicting success on the LTM task. We interpreted that finding as reflecting semantic processes that facilitate storage over longer delay periods (see also Badre and Wagner 2007; Uncapher and Rugg 2005; Wagner et al. 2005). The fact that we now find an overlapping pattern for the early delay stage may be in line with the notion that during this stage an active, dynamic reconstruction of novel information may still be ongoing (Ranganath et al. 2005). In other words, at stimulus offset encoding processes may have been fully completed yet (cf. Bergmann et al. 2013) and participants may still be attempting to form a coherent internal representation in order to help to remember the stimulus set across the delay phase. Note, however, that activation in the inferior frontal gyrus was related to LTM performance in our previous study, but in the current study associated with successful WM. One could argue that the failure of finding differential activity for the LTM contrasts is the result of the relative difficulty of the LTM task. Moreover, the relative difficulty of the WM task may have resulted in additional (semantic) processing. Apart from the inferior frontal gyrus, greater left fusiform gyrus activity was found, also previously being reported to predict LTM success during encoding (Bergmann et al. 2012; Brewer et al. 1998; Wagner et al. 1998; Kirchhoff et al. 2000; Sperling et al. 2003). This has been explained by the fusiform gyrus being involved in the generation of mental images as well as in the processing of deeper high-level perceptual and semantic elements of the memoranda (Dickerson et al. 2007).

Analyses for the probe phase clearly demonstrated greater activation for correct versus incorrect trials in several regions. First of all, greater hippocampal activation was associated with a correct decision on the WM task. Previous work suggested that the hippocampus is part of a generic “retrieval success network,” commonly activated in episodic memory retrieval tasks (Buckner et al. 2008; Henson et al. 2005; Huijbers et al. 2010; Wagner et al. 2005). The fact that we obtained hippocampal activation in our study may reflect the necessity of actively retrieving the to-be-retained information in this rather complex spatial WM task, thereby “mimicking” episodic memory retrieval characteristics (see Bergmann et al. 2015, for a more detailed discussion on this issue). Moreover, as we did not obtain hippocampal activation in a previous study in which we used a non-spatial associative WM task (Bergmann et al. 2015), the strong allocentric nature of the current task may have enhanced the hippocampal activation during retrieval even further. Future studies would have to determine the exact role of the hippocampus during the WM retrieval (see also Schmidt et al. 2007, for a discussion of the role of the medial temporal lobe in allocentric working memory tasks). In addition, we found bilateral insula as well as bilateral post-central gyrus activity associated with a correct decision on the WM task. Although both regions are not typically described as being part of the retrieval success network, previous studies found remarkably similar activation patterns in a visual memory task during retrieval (Sterzer and Kleinschmidt 2010; Abe et al. 2013). This insular activation during visual memory tasks in particular may be explained by the role of the salience network. That is, the salience network (that includes the anterior insular cortex) may have a signaling function to other functional networks that facilitate access to working memory resources (Menon 2015).

### Long-term memory task

Unfortunately, the subsequent LTM effect did not reveal any differential activity for correct versus incorrect trials for any of the four stages, standing in stark contrast to a number of previous reports that typically find encoding-related differential activity (for a review see Kim 2011). This could be the result of the relative difficulty of the LTM task. The distribution of responses as depicted in Fig. 3, for instance, shows that participants had some trouble differentiating between old and new configurations. In our previous study (Bergmann et al. 2012), participants responded with a “6” in only 1.5 % of the non-match cases. In the present study, however, this proportion was 9.8 %.^1^ This may explain why we were unable to detect differential activity between correct versus incorrect trials.

## Conclusion

The present study investigated the neural substrates of successful WM and LTM in an allocentric spatial (object-location) delayed-match-to-sample task. Due to the unexpected (also when compared to previous pilot data) low accuracy on the LTM task, no differential activation could be detected for the LTM task. Nevertheless, the employed paradigm of a combined WM and LTM tasks appeared to be fruitful in our previous two studies (Bergmann et al. 2012, 2015). Future studies investigating the neural substrates of successful spatial WM and LTM need to attempt to lower the difficulty of the LTM task (e.g., by using a more fine-grained confidence interval, potentially leading to better discrimination scores at the highest confidence ratings). Also, the present study highlights the importance of replication studies in the fMRI research field, as we could not replicate all findings of previous research (see also Bennett and Miller 2010, for an extensive discussion). Nonetheless, the present study yielded interesting insights into which brain regions support an accurate WM decision during a spatial WM task, correcting for LTM performance. We found additional evidence for the proposed distinction between early and late stage of the WM maintenance phase and that during the former participants may still be engaged in the active (semantic) construction of an internal representation.

## Footnotes

1 Extensive and careful piloting of this paradigm outside the scanner resulted in an optimal behavioural performance for the working memory and the subsequent memory tasks. However, the behavioural performance of the participants in the present fMRI study was unexpectedly lower.
